# Machine Vision Systems in Precision Agriculture for Crop Farming

**DOI:** 10.3390/jimaging5120089

**Published:** 2019-12-07

**Authors:** Efthimia Mavridou, Eleni Vrochidou, George A. Papakostas, Theodore Pachidis, Vassilis G. Kaburlasos

**Affiliations:** Human-Machines Interaction Laboratory (HUMAIN-Lab), Department of Computer Science, International Hellenic University (IHU), 57001 Thermi, Greece; emavridou@teiemt.gr (E.M.); evrochid@teiemt.gr (E.V.); pated@teiemt.gr (T.P.); vgkabs@teiemt.gr (V.G.K.)

**Keywords:** machine vision, precision agriculture, agrobots, intelligent systems, industry 4.0

## Abstract

Machine vision for precision agriculture has attracted considerable research interest in recent years. The aim of this paper is to review the most recent work in the application of machine vision to agriculture, mainly for crop farming. This study can serve as a research guide for the researcher and practitioner alike in applying cognitive technology to agriculture. Studies of different agricultural activities that support crop harvesting are reviewed, such as fruit grading, fruit counting, and yield estimation. Moreover, plant health monitoring approaches are addressed, including weed, insect, and disease detection. Finally, recent research efforts considering vehicle guidance systems and agricultural harvesting robots are also reviewed.

## 1. Introduction

Precision agriculture is a farming management concept based on monitoring, measuring, and responding to variability in crops. The research on precision agriculture aims to define a decision support system for farm management by optimizing the returns on input while preserving resources. Machine vision has been widely used to support precision agriculture by providing automated solutions to tasks that are traditionally performed manually. Manual methods tend to be tedious and error prone. Machine vision can provide accurate and efficient solutions to support agricultural activities. Moreover, machine learning algorithms enable the analysis of massive volumes of data quickly and accurately, providing a means for the implementation of machine vision applications in agriculture.

Due to the recent advancements in machine vision applications in agriculture, there are several reviews that have been performed focusing on this subject [1,2,3,4,5,6,7,8,9,10,11]. The state-of-the-art published in the last decade pertaining to computer vision for the automatic detection and classification of fruits is reviewed in [1]. A review of 40 research efforts that employed deep learning techniques for agricultural tasks is implemented in [2]. A survey on diseases detection in citrus plant leaves and their classification is presented in [3], while a review on the application of computer vision in the production of grains (maize, rice, wheat, soybean, and barley) is conducted in [4]. In [5], a general overview with a detailed description and technical support is provided regarding spectral bands, imaging sensors, optical systems, and geometric visual system arrangement with respect to specific applications in agriculture. A review is conducted in [6] to map the research progress in vehicle automation in North America over the last 15 years. The key elements of the latter review include navigation sensors, navigation planners, vehicle motion models, and steering controllers. The review presented in [7] investigates the research effort, developments, and innovation in agricultural robots for field operations, and the associated concepts, limitations, and gaps. The review conducted in [8] focus on recent innovations in autonomous navigation systems in agricultural environments. The research in [9] provides a short overview of the worldwide development and current status of precision agriculture technologies of the past years. Relevant research in autonomous agricultural vehicles in Japan is also presented in [10]. In [11], machine vision techniques applied to agriculture are reviewed. This review deals with machine vision technologies for image acquisition and their processing and analysis in agricultural automation, focusing on two types of images, namely visible and infrared.

The work presented in this paper aims to include the machine vision techniques in agriculture-related tasks focusing on crop farming. The purpose of this work is to serve as a guide for the researcher and practitioner in the agricultural field providing the latest advancements on machine vision applications in agriculture. To this end, the review conducted in this paper not only focuses on a specific agricultural challenge (e.g., disease detection) or fruit type or plant (e.g., citrus), but rather outlines research conducted in different agricultural activities covering more technical subjects, such as vision-based vehicle guidance systems and autonomous mobile agricultural robots. In particular, the following main issues are to be addressed:

- Plant and fruit detection approaches
- Harvesting support approaches, including fruit grading, ripeness detection, fruit counting, and yield prediction
- Plant and fruit health protection and disease detection approaches, including weed, insect, disease, and deficiency detection
- Camera types used for machine vision in agriculture
- Vision-based vehicle guidance systems (navigation) for agricultural applications
- Vision-based autonomous mobile agricultural robots

In order to consider any automated precision agriculture engineering design, it would be useful for researchers to be able to find all recent effective machine vision techniques implemented for the broader categories of agricultural tasks, from the out-field development of simple segmentation algorithms, through the in-field integration of sophisticated algorithms, to automated vehicles. Thus, regarding the above considerations, this work addresses the above-mentioned six main issues that need to be considered before the appropriate machine vision system is selected to be installed onboard an agricultural robot for specific tasks in fields. The proposed review on machine vision techniques contributes towards the design of a (semi-)autonomous robot harvester in precision farming applications as part of a national project entitled “Personalized Optimal Grape Harvest by Autonomous Robot” that is being acknowledged.

This work is compiled according to the defined process for conducting reviews based on Kitchenham [12]. Articles reviewed were limited to those published in English. The used search terms were “machine vision in agriculture” and “agricultural robots” which were keyed in GoogleScholar engine, with a recent publication date after 2017. This search initially resulted to 229 papers, from which some of them were discarded due to several reasons e.g., absence of a clear methodology, not enough reference list, publishing houses of low reputation etc. The selected studies should report on the use of machine vision in agriculture for corps farming and the machine vision-based robots in agriculture in the defined time-range. This is the Benitti [13] approach. However, this work focuses on the results of the review, rather than on the approach to conducting the review. 

Plant and fruit detection approaches are presented in Section 2, which is a critical task for performing agricultural activities automatically, such as harvesting, yield estimation, and disease detection. Machine vision approaches for supporting harvesting are reviewed in Section 3, including fruit grading, ripeness detection, fruit counting, and yield estimation. In Section 4, plant and fruit health protection and disease detection approaches are discussed. Specifically, machine vision approaches for weed detection, insect detection, disease and deficiency detection are presented. In Section 5, the different camera types that can be used are presented, aiming to help researchers and practitioners alike in deciding on the equipment to use in each task. Vision-based vehicle guidance systems (navigation) for agricultural applications are reviewed in Section 6. In Section 7, vision-based autonomous mobile agricultural robots are discussed. Lastly, the main findings of this analysis are discussed in Section 8, and conclusions are summarized in Section 9. 

## 2. Plant and Fruit Detection Approaches

The role of image segmentation in agriculture is the separation of the plant from the background and/or the fruit from the rest of the plant and the background. This task is a key requirement for performing agricultural activities automatically like harvesting, yield estimation and disease detection. Image processing technologies can conduct object detection accurately, quickly and non-invasively, i.e., non-destructively for the plants and fruits. Image processing for plant and fruit detection can be challenging for several reasons. First, different lighting conditions can cause the color degradation of the target’s appearance. Thus, segmentation based on color information can be seriously affected by illumination. The segmentation problem becomes even harder when the target has a similar color to the background. For example, when the target is a green fruit, like an apple or grape, it is difficult to separate the fruit from the background containing leaves and branches which also have a green color. In those cases, segmentation based on color does not provide good results. To this end, methods that deploy different types of features, such as texture [14] and shape, and examine a group of pixels and their relations to each other have been proposed. However, many approaches require the usage of thresholds for the features’ (e.g., color, shape or size) values that need to be defined for any different image, leading to threshold-dependent performances. It is obvious that the overall accuracy of fruit and plant detection is highly dependent on image segmentation performance. Therefore, a robust segmentation algorithm is needed, displaying high accuracy regardless of the fruit color or the surrounding environment. Thus, due to the varying challenges, a literature review of recent research efforts in plant and fruit detection is considered to be of great importance. In what follows, relevant recent methodologies of the bibliography are presented. 

A grape identification method based on an artificial neural network (ANN) and a genetic algorithm (GA) using color features is presented in [15]. A GA is employed to optimize the ANN structure and select superior color features simultaneously. Although this method performs well for a specific task, i.e., for mature grapes, it does not have the same performance when the color of the fruit is similar to the rest of the plant. This is the main disadvantage of segmentation algorithms that use color features. In order to perform well, a higher contrast is needed between the target and background. An approach that considers the aforementioned disadvantage is presented in [16]. The proposed algorithm can detect green apples in natural scenes where the background has a similar color to the targets. Firstly, the image is represented as a close-loop graph with superpixels as nodes. Then, these nodes are ranked based on their similarity to the background and foreground queries in order to generate the final saliency map. Finally, Gaussian curve fitting is carried out to fit the V-component in YUV color space in salient areas, and a threshold is selected to binarize the image. This method displays poor performance in segmenting the edges with shadows and intense facula around the object. Moreover, the performance of the method is threshold-dependent, yet easily affected by varying lighting conditions.

An alternative method developed to perform under realistic and uncontrolled conditions is proposed in [17]. The method is tested to detect apple flowers. The generalization capability of the proposed approach on additional datasets is also examined. The method consists of three stages: (i) computation of region proposals, (ii) feature extraction using a fine-tuned Convolutional Neural Network (CNN) based on Clarifai architecture, and (iii) the application of Principal Component Analysis (PCA) to reduce the feature dimensionality and classification of each region according to the presence of flowers with a Support Vector Machine (SVM) classifier. Among the research findings of this work is that color analysis alone leads to limited applicability in scenarios involving changes in illumination. The technique presented in [18] is based on both color and texture features in order to detect cucumbers in a greenhouse. SVM is used for the identification task. Deep learning techniques currently represent the state-of-the-art for computer application systems. However, a large amount of both data and computational power are required to meet the expectations of better performances. Moreover, uneven illumination on the cucumber surface and the irregular growth of fruit obstructed the segmentation task. To detect fruits/plants of different sizes, multiscale algorithms for multiple fruit/plant sizes need to be developed. An image segmentation approach based on color, texture and shape is presented in [19]. This approach applies Otsu thresholding on hue, saturation, intensity (HSV), and luminance, chromaticity blue, chromaticity red (YCbCr) color space for mango leaves. Segmentation results are promising, yet they are also threshold-dependent and unstable under varying illumination. 

The aforementioned drawbacks, i.e., different plant/fruit sizes and varying illuminance, are addressed in [20]. The intensity of sunlight changes from morning to night, inducing change in color features. Therefore, the segmentation system needs to be trained for all the conditions so as to be applicable for real-time use during all day. In [20], a new color space in field conditions for segmenting green plants from their background is suggested. The algorithm can perform well under different light intensities and with different crop growth stages. The suggested machine vision system is comprised of two steps: the first step consists of identifying the color conditions and the second step includes segmentation using a hybrid artificial neural network-harmony search (ANN-HS) classifier. A computer vision system for the classification of wheat grains to their species using a multilayer perceptron (MLP) ANN is proposed in [21]. Images are converted to grayscale, binarized using the Otsu method and segmented using the thresholding operation. The properties of size, color, and texture are captured for each grain, and are given as input to the classification model. The main disadvantage of the method is that grain images are captured in artificial lighting (strip LED lighting) and not refer to real environmental lighting conditions.

A flower and a seedpod detection method in a crowd of soybean plants under completely outdoor environments is presented in [22]. The detection of flower regions is performed using image segmentation by SLIC (simple linear iterative clustering) [23], which conducts pixel clustering based on the hue similarity and the distances between two pixels. A CNN is introduced to make the final decision for flower detection. For flower counting, features from an accelerated segment test (FAST) are used for all the detected flower regions [24] to find saliency features (i.e., key points) from the surrounding regions of the detected flowers. Then, Oriented FAST and Rotated BRIEF (ORB) features [25] are calculated for all the key points and find the matched points in the two consecutive pictures in order to avoid counting the same flower twice, due to overlapped regions. For detecting soybean seedpods, the Viola-Jones object detection method [26] is adopted. Contrast limited adaptive histogram equalization (CLAHE) [27] is performed to enhance the robustness against various lighting conditions. Finally, CNN [28] is used to predict which of the candidate regions are seedpod parts. Although the method returns good performances, open issues still remain. Such issues include the flowers of other plants located behind a target plant sometimes being detected together, and stems and leaves sometimes wrongly being detected as seedpods, and there are several cases whereby seedpods are wrongly detected 

An image segmentation method for rice panicles in the field is implemented in [29]. First, Simple Linear Iterative Clustering (SLIC) [23] is used to generate candidate regions. Then, a CNN [30] is applied as a candidate region classifier. Finally, the entropy rate superpixel (ERS) [31] algorithm is developed for optimizing the segmentation result. Compared with other segmentation approaches, namely HSeg (hue plane threshold segmentation) [32], i2 hysteresis thresholding [33], and jointSeg [34], the proposed Panicle SEGmentation (Panicle-SEG) algorithm shows higher segmentation accuracy. Parallel computing and deep learning with graphical processing unit (GPU) acceleration is adopted. The algorithm runs comparatively faster, however is still not suitable for real-time applications. A cotton leaf segmentation method based on an immune algorithm (IA) and pulse coupled neural networks (PCNN) is presented in [35]. Three anti-light color components are selected by histogram statistical with mean gray value. Then, the optimal parameters of PCNN model and the optimal number of iterations are determined by using IA optimization so that PCNN model can segment the objects in cotton regions more effectively. Four image segmentation methods, namely the Otsu algorithm, K-Means algorithm, FCM algorithm, and PCNN, are compared with the proposed method to show that it has good resistance to light changes and complex backgrounds. However, the disadvantage of this method is the dependence on the iteration step, and its real-time performance is relatively poor. Meanwhile, weeds with a similar color to cotton may also be detected as the target region. An image segmentation method of overlapping leaves based on the Chan-Vese (C-V) model and a Sobel operator is implemented in [36]. First, the background is removed using a threshold with respect to the relative levels of green in the image. The contour of the target leaf is extracted using the C-V model. Then, eight directional Sobel operators are applied to detect the edges of the leaf. Finally, the overlapping target leaf is extracted by combining the results obtained by the C-V model and the Sobel operator. The method needs to be extended to different types of crop, and improved in terms of robustness against environmental conditions. 

Table 1 summarizes the main characteristics of recent methods of the literature for plant and fruit detection. In the Discussion section, additional information on the literature is provided regarding research gaps, pros and cons, and potential future directions.

Table 1 reveals the need to analyse the fruit/plant images in an appropriate color space in order to better detect the fruit/plant by extracting discriminative color-based features. Moreover, from the contents of Table 1, it is deduced that the CNN models do not show the highest accuracy in all cases, but there are shallow models (e.g., MLP, SVM) that perform well in some cases where the available data are limited.

## 3. Harvest Support Approaches

Harvesting is a critical agricultural activity which involves the gathering of ripe crops or fruit from the fields. Intensive labor is required for detecting and collecting the mature crop/fruit. A manual process of sorting is then performed based on various characteristics like size, maturity level, shape, and damage level. However, manual sorting is time consuming and subject to human errors, leading to variability in the quality of the final product [35]. Thus, there is a need for more accurate and efficient methods for evaluating the fruit or crop collected. Sorting agriculture products automatically is more efficient compared to the manual approach, which is slow, tedious, and error prone. Fruit counting is another very important task for supporting harvest. An accurate and automated fruit counting method could assist farmers in optimizing their harvest process. A better understanding of the variability of yields across fields can help growers to make more informed and cost-effective decisions for labor allotment, storage, packaging, and transportation [37]. 

Machine vision has been widely used for automating the harvest process allowing faster and accurate sorting and counting of fruit without the use of intensive labor. However, there are many factors that make those tasks difficult and challenging. Different lighting conditions can cause variation in the color of the fruit, leading to misclassification. In case where the fruit has a similar color to the rest of the plant (like green apples), the task becomes even more challenging, making it hard to detect them using color information. Moreover, overlapping fruits make the detection harder and can lead to misclassification and miscounting. In what follows, recent approaches for the support of harvest are presented. Since harvesting is about detecting and collecting the mature crop/fruit, the selected approaches of the literature are divided and presented into two relevant sections: Section 3.1 presents fruit grading and ripeness detection approaches, while Section 3.2 deals with fruit counting and yield prediction. Section 8 summarizes the presented methods of this section, by providing the research gaps, pros and cons, and potential future directions for both approaches.

### 3.1. Fruit Grading and Ripeness Detection

Fruit grading refers to the sorting of fruit based on parameters like size, shape, and maturity level. Manual fruit grading is labor intensive and leads to errors due to human involvement in the sorting process. To this end, machine vision is used for the automated and accurate grading of fruit.

A mango grading approach is proposed in [38]. An image processing algorithm based on region global thresholding color binarization, combined with median filter and morphological analysis is developed to classify mangos into one of three mass grades (large, medium and small). The algorithm is simple and can be used in real-time applications. Moreover, it is general in nature and with proper adjustment can be applied to other crops as well. However, fluorescent light may be efficient for experimenting, yet real-field applications need algorithms that are able to perform well outside of the laboratory. An approach on mango size estimation is presented in [39]. A cascade classifier with histogram of oriented gradients (HOG) features is used. Then, Otsu’s method, followed by color thresholding is applied in the CIE L*a*b* color space to remove background objects (leaves, branches etc.). A 1D filter is developed to remove the fruit pedicles, and an ellipse fitting method is employed to identify well-separated fruit. Finally, fruit lineal dimensions are calculated using the RGB-D depth information, fruit image size, and the thin lens formula. This method is suitable for rapid in-field fruit size estimation, practical in terms of cost and ease of use, but cannot be used in direct intense sunshine.

A methodology to estimate the maximum/minimum (polar/equatorial) diameter length and mass of olive fruits by means of image analysis is presented in [40]. Image segmentation is performed based on mathematical morphology, which maximizes the contrast between the olives and the background. Then, the olives are segmented by automated thresholding based on statistical bimodal analysis. Results underscore the robustness and accuracy of the method. However, future trials in different lighting systems need to be investigated in order to verify the generality of the method to other cultivars. In [41] a method for detecting the maturity levels (green, orange, and red) of fresh market tomatoes (Roma and Pear varieties) is presented. The proposed technique combines the feature color value with the backpropagation neural network (BPNN) classification technique. Results indicate that the classification based on color features can potentially be applied for the in-field yield estimation of fresh tomatoes. Yet, when the maturity of the tomatoes is green, identification results range within a considerable margin of error. 

A soya bean ripeness and health detection method based on color and texture features is proposed in [42]. The input images are converted from an RGB to HSI color model. For extracting the texture features, a gray level cooccurrence matrix is used, and statistical parameters such as energy, contrast, correlation, and homogeneity are extracted. Lastly, an ANN is trained based on the extracted features for the classification into been ripe, unhealthy, leaf, unripe or ripe but not ready for harvest. Though the use of soya beans differs around the globe, predicting their ripeness period will help reduce losses. The used images are captured under an uncontrolled environment to obtain their natural color without introducing any variation in intensity/brightness to the images. 

An analysis of the performance of RGB, NIR, and depth images for immature citrus fruit detection using machine vision techniques is conducted in [43]. First, circular object detection is performed to find potential fruit areas. For this purpose, circular hough transform is used for RGB and NIR images and CHOI’s circle estimation (‘CHOICE’) algorithm is developed for depth images. The ‘CHOICE’ algorithm looks for the center of the circles that have the maximum divergence and vorticity values. Moreover, the boundary of the circle is chosen according to the sign of the divergence and vorticity values. This way, the CHOICE algorithm can find possible spherical objects in the depth images. Lastly, the classification of citrus fruit from the background is implemented using AlexNet convolutional neural network [44]. The results of this study can play a significant role in evaluating the most efficient image types in an outdoor machine vision systems for citrus yield prediction. The proposed algorithm can be improved by adopting a tolerance factor to ignore noise from sunlight. Moreover, it can be implemented in real-time and combined with unmanned aerial or ground vehicles to have a fully automated citrus yield prediction system.

Table 2 summarizes the main characteristics of the recent fruit grading and ripeness detection approaches of the literature. 

From the contents of Table 2 it is deduced that the fruit grading and ripeness detection are two quite easy tasks since the reviewed works solved these problems with high accuracy rates (above 90%). However, a more careful analysis of the above methods reveals that these tasks are not so difficult for the cases of the examined fruits (mango, olives, tomato, citrus) since their shape is very clear and with large variations, while their ripeness can be easily measured visually by processing their color changes. However, there are cases where the shape of the fruit is not so clear, or with small variations due to overlaps, and their ripeness is not indicated so much by color changes, as in the case of green grapes [14].

### 3.2. Fruit Counting and Yield Prediction

Fruit counting is important for estimating the yield. The estimation of fruit count using machine vision is quite challenging. Counting the number and measuring the size of fruit by machine vision is based on the suggestion that all fruit on a tree can be seen and are not obstructed by leaves. The scientific challenge is to identify each fruit in the tree image with some fruit hidden within the canopy, especially in the early period. The appearance of fruit may vary due to illumination and occlusion caused by the surrounding foliage and fruits, leading to miscounting. However, early prediction is essential for planning labor, bins, and harvest management as well as transport, grading, and storage. Recent research efforts which address the problem of fruit counting are presented in this section.

A methodology for the detection and counting of marigold flowers using HSV color space for segmenting the flowers from the background and fitting with circular hough transform (CHT) for counting is presented in [45]. The advantage of this approach is that it is capable of detecting and counting marigold flower even in occlusion and/or overlapping conditions. For better results, high resolution cameras and high-quality images are required. In [46], a fruit detecting and counting approach is implemented based on a CNN. A counting algorithm based on a second convolutional network is then used for the estimation of the number of fruits and a linear regression model for mapping what fruit count estimates to a final fruit count. The advantage of this approach is that it addresses the issue of overlapping fruit. However, it is susceptible to errors due the human-generated labels (ground-truth) for the input data. In [47], a method for citrus detection and counting is proposed. The method consists of the following steps: conversion of RGB image to HSV, thresholding, orange color detection, noise removal, watershed segmentation, and counting. The watershed transform is effective for the segmentation of objects with unclear boundaries. However, it can cause over-segmentation due to the presence of many local minima [31]. For that purpose, marker-controlled watershed transformations are deployed. A disadvantage of this approach is that fruits could be detected twice due to their areas being split by branches or leaves.

An image analysis algorithm to detect and count yellow tomato flowers in a greenhouse is implemented in [37]. This method uses an adaptive global threshold, segmentation over the HSV color space, and morphological cues. The adaptive threshold divides the images into darker and lighter regions. Then, segmentation on the HSV color space is performed, and classification is made according to the size and location of the flowers. The main advantage of this method is that it considers the varying illumination conditions. However, images taken in the afternoon from an angle facing the plants provide better results in terms of precision and recall than any other angle. An approach of using image analysis and tree canopy features to predict early yield with ANNs is presented in [48]. Tree canopy features are proposed in order to overcome the difficulties of counting when leaves cover the apple fruit. Two back propagation neural network (BPNN) models are developed (for the early period after natural fruit drop in June and the ripening period). For each sample image (canopy image), pixels are segmented into fruit, foliage, and background using image segmentation. Four features are extracted from the images, namely total cross-sectional area of fruits, fruit number, total cross-section area of small fruits, and cross-sectional area of foliage. Then, a BPNN is deployed to learn their mutual relationship in order to predict the actual weighted yield per tree. The proposed method may reduce the adverse influence from foliage. Hence, the method could be used for apple yield prediction in early and late growing periods. However, all experiments were conducted on a general model of tree form. Further research will focus on separating a site-specific model from the general model, and investigate the adaptation of the algorithm to other tree forms or similar fruits.

Table 3 summarizes the main characteristics of the recent fruit counting and yield prediction approaches of the literature.

Table 3 indicates that fruit counting constitutes a difficult task, mainly due to the complex background generated by the presence of the tree leaves, which increases the overlapping regions. Although the color features can help with fruit detection, the accuracy of fruit counting is highly dependent on the size of the fruit.

## 4. Plant/Fruit Health Protection and Disease Detection Approaches

The health of plants and fruits can be affected by various factors. One of most important factors that can affect the fruit and plant health is weeds. Weeds require nutrition, light, and moisture, which are shared with the growing plant. Thus, if a weed is not detected and removed early it will have a negative impact on the growth and quality of the final product. Machine vision has been widely used for automating weed detection. This task becomes very challenging when there is not significant difference in the color between the plant and the weed. Another factor that can have a negative impact in the health of a plant is insects. The monitoring of insects automatically can help growers to protect plants and fruits. Machine vision has also been used for detecting diseases and deficiencies. There is a considerable body of literature regarding disease detection [5]. Disease detection is a challenging task, mainly due to the variability in color and texture of the diseased part. Changing lighting conditions can also affect disease detection approaches significantly [3]. 

The aim of this section is to provide insight into the recent machine vision algorithms that address the aforementioned factors affecting the health of plants. Thus, recent research efforts using machine vision in (1) weed detection, (2) insect detection, and (3) disease and deficiency detection are briefly presented. In the Discussion section, additional information on the literature is provided, regarding research gaps, pros and cons, and potential future directions for all three tasks.

### 4.1. Weed Detection

Weed detection refers to the task of automatically detecting weeds between crops. Weeds can affect the growth of the plant and the quality of the final product. The challenge in this task is to accurately discriminate weed from plant. The presence of illumination variations, overlapping leaves, and insignificant color differences between weed and crop make this task more challenging.

In [50], a weed detection approach is implemented based on HSV color space for the image segmentation and a CNN for the classification to weed and carrots. Apparently, due to the great difference of color between carrots and weeds, this method based on color performs well. Color features are most effective for crops with a distinguishable and unique color [42]. Consequently, in cases whereby the color difference is not so significant, it is very possible that this method could not accurately discriminate weed from crop. Moreover, color-based methods are susceptible to different lighting conditions. 

For that purpose, a texture-based approach to segment the weeds from the main crop based on wavelet texture features and ANNs is presented in [51]. Wavelet transformation extracts information from several spatial orientations which is helpful for analyzing the content of images, texture discrimination, and fractal analysis [52]. Principal component analysis is also used for transforming the feature space to a lower number of dimensions. Experimental results reveal that the proposed approach is able to distinguish weed from crop even when there was significant amount of occlusion and leaves overlapping. Errors occurred mainly in areas where the crop’s leaf surface was wrinkled or deformed. However, these errors were very small, accounting for less than 1% of the misclassifications. A weed detection approach from video streams is proposed in [42]. The HSV color space is used for discriminating crop, weeds and soil. The region of interest (ROI) is defined by filtering each of the HSV channels between certain values (minimum and maximum threshold values). The region is then further refined using a morphological erosion and dilation process, and the moment method is applied to determine the position and mass distribution of objects in video sequences so as to track crops. High accuracies are obtained in the classification of various categories of crop leaves.

A weed recognition system is proposed in [53] to be used in a robotic system for the weed detection problem. It uses an image resolution of 200 × 150 pixels, speed-up robust feature (SURF) [54] features over dense feature extraction, an optimized Gaussian Mixture Model based codebook combined with Fisher encoding, a two level image representation, and a linear classifier. The method stands out for its simplicity of computation, leading to an improved speed of execution. However, the possibilities of further improvements to the speed of execution using advanced parallel and GPU based computing methodologies needs to be investigated. Moreover, the dataset acquisition process needs to be extended in order to better represent realistic working conditions in various environmental and illumination conditions. A weed detection approach that relies on a fully convolutional network with an encoder–decoder structure incorporating spatial information by considering image sequences is presented in [55]. The experimental results show that it performs better compared with recently published approaches: (i) using a semi-supervised vision and geometry based approach based on random forests [56] and (ii) employing a purely visual FCN classifier solely based on RGB data [57], which takes vegetation indices as additional plant features (PF) into account. Results reveal that the proposed system generalizes well previously unseen fields under varying environmental conditions, without retraining of the model. A vegetation segmentation and classification method is proposed in [58]. First, the normalized difference vegetation index (NDVI) images used as training data are represented by the max-tree hierarchy [59]. The segmentation step selects regions from the hierarchy based on their size stability over a series of grey levels. In contrast to the global thresholding methods like Otsu’s thresholding [60], a decision about each region is taken locally. This way, even in the presence of noise, the plant regions are easily visually distinguishable due to their high local dissimilarity with their background. The segmentation process results in a set of distinct regions with associated attributes, which are used as region features for the training of an SVM classifier. Although the advantage of the region-based approach is that it only classifies a few objects per image, it cannot accurately classify the pixels of regions that contain both value crop and weed pixels. For those cases, a pixel-based classifier is proposed to be investigated in future work.

Table 4 presents the main characteristics of recent weed detection approaches of the literature.

From Table 4 it is obvious that the problem of weed detection can be solved with high accuracy only in the cases where the fruit/plant differs significantly in color to the weed, e.g., carrots. On the contrary, when the color of the fruit/plant is very close to the color of the weed, more sophisticated features (e.g., texture features) need to be deployed in order to detect accurately the weed.

### 4.2. Insect Detection

Pest management is one of the main concerns for farmers. Reductions in production loss and crop damages can affect marketable yields. Therefore, farmers use several methods to control and protect fields against pest damages. During recent years, the use of pesticides has increased due to their initial low cost, easy accessibility, quick influence, and the lack of knowledge on the part of farmers, resulting in dangerous consequences for public health, animals, and the environment. Thus, better methods of pest control, other than the use of chemical pesticides, are needed. Insects can cause great damage to plants and fruits. For example, *Lobesia botrana* is an invasive insect considered as one of the most damaging pests in vineyards (*Vitis vinifera* L.) [53]. The traditional methods for insect identification are time-consuming and require expert knowledge. To this end, machine vision has been deployed for automatically identifying insects. Machine vision for automated insect identification, may lead to increasing the work speed and precision, and decreasing human errors, since farmers may have limited pest scouting expertise.

A method for *L. botrana* recognition is presented in [62] using a clustering-based image segmentation with descriptors, which considers gray scale values and gradients in each segment. The proposed system allows for fast and scalable cloud-computing analysis of the images, providing an ideal environment for in-field applications. In [63], a grapevine bug detection method is implemented. The method involves the use of scale-invariant feature transform (SIFT) for calculating low-level features, bag of features (BoF) for building an image descriptor, and an SVM classifier. This method claims to be robust to realistic scenarios, i.e., in outdoor, under natural field conditions, in winter, without artificial background, and with minimal equipment requirements. A pest detection method in strawberry plants is proposed in [64]. The methodology uses an SVM model using hue, saturation and intensify color indices, and the ratio of major diameter to minor diameter as a region index. The method is implemented for real-time detection of parasites from flower surface images with relatively small error.

Table 5 summarizes the main characteristics of the above research efforts on insect detection.

From Table 5, it is obvious that the task of insect detection constitutes a typical object recognition problem, which can be addressed by applying shallow machine learning models with acceptable accuracy. However, it seems that it is quite difficult to detect multiple types of insects simultaneously, probably due to the small size of the insects compared to the size of the fruit/plant. 

### 4.3. Disease and Deficiencies Detection

Disease and deficiency detection refers to the automatic detection of parts of the plant that are not healthy. This subject is under research due to its importance and variability, a fact that makes this task quite challenging. Depending on the kind of disease or deficiency, and the type of fruit or plant, the diseased part varies in color and texture. Changing of lighting conditions can also affect the appearance of the diseased part [3].

The study presented in [65] focuses on the automatic identification of nutritional deficiencies of Boron (B), Calcium (Ca), Iron (Fe) and Potassium (K), using descriptors of the shape and texture of coffee leaves images. Image segmentation is performed using the Otsu method, the blurred form model (BSM) descriptions, and the gray-level co-occurrence matrix (GLCM) to extract shape and texture features. Then, k-Nearest Neighbors (k-NN), naïve Bayes and neural network classifiers are trained by using the extracted features in order to predict the type of deficiency presented in each analyzed image. Although the experimental results show that the naïve Bayes classifier and the BSM descriptor have higher performance than the rest of the methods, the general performances were relatively poor (F-measure 64.9%). An approach for disease detection in citrus plants is presented in [66]. Delta E (DE) is used for segmentation, RGB, HSV (color histogram) and LBP (textural information) as descriptors on the collected images. Fine k-NN, Cubic SVM, boosted tree, and bagged tree ensemble classification methods are used, with the bagged tree ensemble classifier performing better by using any color features compared to the other classifiers. The experimental results showed that the color features are important as well as the textural features in the detection of plant diseases. Further, the combination of these features is also useful in the detection of the diseased area in the plant. A correlation-based feature selection method to identify apple leaf disease is proposed in [67]. Color, texture, and shape features are used as inputs to an SVM model to classify apple leaf diseases. However, the proposed method fails to identify apple leaf diseases under natural illumination. In [68], a method for plant disease detection based on SIFT features is proposed. The input image is pre-processed to extract the whole region of the leaf. the scale-invariant feature transform (SIFT) features are extracted from the pre-processed image. The extracted SIFT features are modeled by a generalized pareto distribution (GP). The two parameters of the GP model are combined with GLCM statistical features to form the new proposed feature vector. Finally, an SVM classifier is trained using the extracted feature vectors. The proposed features achieve acceptable classification accuracy. However, although the training time and prediction speed of the classifiers may be acceptable for mobile devices, but not applicable for real-time prediction.

A method for detecting lesions on wheat leaves based on the C-V model [69] is presented in [70]. The C-V model is used in agricultural image processing, where the data contents of a region of interest are extracted as prior information. However, in the C-V model the R, G, and B channels in the region of interest are fixed, and the weights for each channel of the color images are determined artificially. In this paper, PCA is used for selecting three color channels from the R, G, B, H, S, and adaptive weights of each channel are obtained by calculating the contrast of the object and background pixel values. K-means clustering is then used for separating the lesion region from the rest of the leaf. Experimental results show that this method achieves better segmentation accuracy than those of the initial circle curve or initial Otsu segmentation with fewer iterations. However, when the examined regions are ambiguous with respect to the background, the proposed method does not achieve satisfying results.

In [71], a content based image retrieval system (CBIR) for retrieving diseased leaves of soybean is presented. CBIR based system involves two steps; feature extraction and feature matching. For this purpose, color, shape and texture features are extracted. Specifically, color features are extracted using HSV color histogram. Shape features are provided in the form of matching key points by the SIFT algorithm. Finally, a new texture feature is proposed, namely local gray Gabor pattern (LGGP), by combining local binary pattern (LBP) and Gabor filter. The extracted feature vectors are stored and when a query image is given, its features are extracted, and its distance to the feature vectors is calculated in order to find the one with the smallest distance. The proposed system provides promising results. These results are database dependent. If size of database is changed then result may alter. A method for detecting diseases in potato leaves is implemented in [72]. Leaf is segmented from the background with the determination of thresholds for L*, a* and b* channels instead of using auto-threshold like Otsu. Then, the GLCM is applied for extracting statistical texture features like contrast, correlation, energy and homogeneity. Numerical indicators like mean, standard deviation, entropy, skew and energy are calculated from the histograms of the extracted color planes. Finally, an SVM classifier is trained on the extracted features to classify the leaves into affected and healthy ones. The classification rates of the method are high, pathing the way toward automated plant diseases diagnosis on a massive scale. Potentially, more types of diseases affecting various types of plants will be integrated to the system.

An image processing method using color information and region growing for segmenting greenhouse vegetable foliar disease spot images captured under real field conditions is presented in [73]. The quality of color index-based segmentation decreases dramatically when locally uneven illumination occurs. For that purpose, a comprehensive color feature (CCF) is proposed which combines excess red index (ExR) [74], H component of HSV color space, and b* component of L*a*b* color space, which are invariant to illumination [75]. Then, an interactive region growing method based on the CCF map is used for disease spot segmentation from the clutter background. The experimental results indicate that the algorithm performs better than K-means clustering and Otsu algorithm for the segmentation of disease spots on images captured under real field conditions. Both the K-means clustering algorithm and Otsu algorithm are affected by locally uneven illumination and background noise, causing a lot of false positives segmentations. A method for the detection and classification of diseases in citrus leaves is implemented in [76]. Image segmentation is performed with K-means clustering. GLCM texture features are extracted and used for training an SVM classifier for the disease classification. Results are promising, and future work includes the experimentation of diseases in different plant species. In [77], a disease detection and classification method for citrus leaves and fruit is presented. First, image pre-processing is performed to improve the contrast of input images by applying a top-hat filter and Gaussian function. Specifically, the top-hat filter is used to improve the visual quality of input image, which is further improved by a difference-Gaussian image. For the segmentation of the disease lesion spots a weighted segmentation method is proposed, which uses chi-square distance and threshold function. Then, color, texture, and geometric features are fused in a codebook. Feature selection is performed by a hybrid feature selection method, which consists of PCA score, entropy, and skewness-based covariance vector. The selected features are given as inputs to a multi-class support vector machine (M-SVM) for citrus disease classification. A comprehensive comparison shows that the proposed method outperforms the existing methods in the literature when tested on the same datasets. Future work includes a deep model to be applied to the selected citrus datasets, as the deep learning is reported to perform well in the field of computer vision. However, the latter needs a big dataset in order to be implemented.

A cucumber disease recognition approach is proposed in [78]. The method consists of segmenting diseased leaf images by K-means clustering, extracting shape and color features from lesion information, and classifying diseased leaf images using sparse representation (SR). An advantage of this approach is that the classification in the SR space can effectively reduce the computation cost and improve the recognition performance. Although the SR based recognition method shows its advantage over other methods, at the current stage it is not clear how to efficiently construct the over-complete dictionary of SR. A plant disease detection and classification method based on fusion of super-pixel clustering, K-means clustering, and pyramid of histograms of orientation gradients (PHOG) algorithms is proposed in [79]. First, the images are divided into a few compact super-pixels by super-pixel clustering algorithm (after transformation to L*a*b* color space from RGB). Then, K-means clustering is used to segment the lesion image from each super-pixel. Finally, the PHOG [80] features are extracted from three color components of each segmented lesion image and its grayscale image. The concatenated four PHOG descriptors are given as inputs to a context-aware support vector machine (C-SVM) classifier with a radial basis kernel function for the plant disease classification. The effectiveness of applying super-pixels to parameter estimation of the algorithm is because they reduce the complexity of images from thousands of pixels to only a few hundreds of super-pixels.

Table 6 summarizes the main characteristics of the discussed research efforts on disease and deficiencies detection.

The problem of disease and deficiencies detection seems to be a difficult task as can be concluded from Table 6. The accuracy of the reviewed methods is mostly limited, below 90%, with almost of them using texture features and shallow machine learning models. The difficulty of this problem comes from the need to describe the diseased surface of the leaves in such a way as to discriminate it from the healthy surface. Moreover, the high overlap regions of the leaves do not permit the accurate quantification of the leaves’ surfaces.

## 5. Camera Types Used for Machine Vision in Agriculture

The selection of the appropriate camera equipment for a specific machine vision application in crop farming constitutes a quite laborious task, considering the diverse cameras that are available in the market. Therefore, some selection criteria need to be defined by the designer considering mainly the application requirements that need to be satisfied. The purpose of this section is to give useful insights to researchers by outlining the general types of equipment used for acquiring images/video for machine vision applications in agriculture according to the literature. The camera’s sensor resolution, frame rate, image transfer rate (connectivity), and price seem to be the most important factors that need to be examined in order to select the appropriate camera that fits the needs of each application. 

Based on the application, the camera device can be decided based on three broader categories, namely: RGB cameras, multispectral cameras and stereovision cameras. The most simple and affordable vision system consists of a single RGB camera. This is the main reason why they are extensively used in agricultural machine vision applications for fruit/plant detection [16,17,22,45,46,47], yield prediction [48], segmentation tasks [21,29,35,36], disease detection [66,69,71,73], ripeness detection [40,41], weed detection [53,58,61] and insects detection [63,64]. As an alternative affordable and convenient solution for image capturing, the RGB build-in cameras of mobile devices are also used [19,37]. When using RGB cameras, the potential for effective image processing is due to the use of efficient image analysis algorithms, rather than image quality. RGB color space is adequate for image display, but not for color processing, since the intensity is not decoupled from the chromaticity and there is a high correlation between the components R, G and B [81]. For this reason, RGB images are not appropriate for algorithms that use color processing. Moreover, it is well-known that the color information in an image is very important in image segmentation, but it is very sensitive to illumination. The negative effects of nonuniform illumination may be reduced by normalizing the color vectors, however, they are not totally eliminated [81]. On top of that, the complex background increases any induced difficulty. The quality of images depends on camera’s intrinsic properties dealing with lighting conditions, sizes of objects, and distance from the object. For better results, cameras with higher resolution are preferred.

Humans perceive colors in the three broad channels R, G, and B, but plants are potentially discriminated by higher-precision color measurements [82]. Some studies utilize multispectral images, referring to hyperspectral, thermal, or ultrasonic camera technologies for object (fruit, plant, insects, leaves etc.) detection [20,38,43,55,83]. These technologies typically provide better results than conventional RGB color images. This is because objects with similar color may exhibit different reflectance in non-visible regions, and thereby, they are easily distinguished with non-conventional RGB cameras. On the other hand, these technologies require expensive devices compared with RGB cameras, and therefore are not affordable for practical use. The combination of the two technologies, i.e., the addition of multispectral filters to a low-cost vision system, has the potential to provide features of interest strongly related to discriminatory wavelengths [82]. 

Furthermore, the differentiation of distinct plants, such as crops and weed, is difficult in a 2D image. As an additional parameter, the dimension of crops (depth information, crop height, leaf shape, leaf area etc.) can be monitored. This can be achieved with stereo vision. Three dimensional plant structure derived from stereoscopic cameras is used in applications to crop and plant monitoring and species discrimination [39,43]. 

The above analyses reveal that there are diverse types of cameras available for image acquisition, depending on the specific agricultural application. However, the critical factor in all cases is the camera’s resolution, which should be high enough to capture the details of the scene especially in the cases of insect and disease detection [71]. Moreover, when there are needs for embodying machine vision capabilities to an autonomous vehicle (agrobot), the usage of an industrial camera [20,55] with high speed communication abilities (e.g., GigE) and increased frame rate is, in some sense, inevitable. 

## 6. Vision-Based Vehicle Guidance Systems for Agricultural Applications

After examining the most representative approaches in machine vision applications for crop farming and the camera equipment used to implement them, it would be very constructive to review briefly the vision-based vehicle systems proposed in the literature that deployed such kinds of machine vision approaches.

Automated agricultural vehicles are capable of a longer duration of work, as an autonomous vehicle may outlast a human worker, increase productivity, increase application accuracy, and operation safety. Autonomously driven robots can help farmers reduce the manpower and hours needed to fulfill the needed agricultural tasks, and at the same time conduct other high-level tasks, as navigation is automated. Moreover, autonomous navigation is environmental and economically friendly compared to traditional methods of accomplishing the same assignment, since vehicles do not wander around the fields meaningless but navigate optimally to specific areas of interest by choosing the shortest paths. Thus, farmers can better use their resources in terms of time and money (i.e., fuel and salaries), therefore saving money and economizing a better product for the masses. With these benefits in mind, autonomous vehicles no longer exist at the fantasy level, but are becoming feasible, and they are much needed nowadays [84]. For these reasons, automatic vehicle navigation in agricultural applications is considered as a separate field of science.

Early guiding systems used sophisticated sensors, demonstrating technical feasibility [85]. High cost and low reliability were the main reasons for non-commercialization of these systems. Nowadays, navigation is strongly related to positioning information of the vehicle in a global or a local coordinate system. Global positioning systems (GPS) are widely used as global guidance sensors [86,87]. The main disadvantage of GPS-based navigation is the low precision of GPS receivers. Typical GPS precision is about ±50cm, which is an important tolerance in the case of precision farming. 

**Figure 1:**
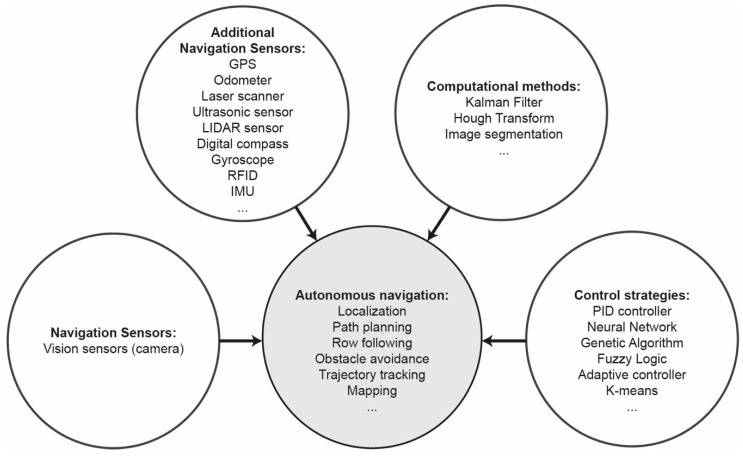
Basic parts of an autonomous vision-based navigation system.

Machine vision is used complimentary to GPS and sensors in order to enhance the precision of navigation. When crop row structure is distinct in a field, machine vision can be implemented to automatically guide a vehicle in the field. On one hand, the guiding sensor (camera) is only local, since it reveals information regarding the relative location of the vehicle. On the other hand, the use of local features can be used to fine-tune the vehicle navigation course [88]. Machine vision provides the technological characteristics to simulate the eyes of a human operator, thus, has a great potential in navigation systems [89]. The problem of navigation in plant structures is the fact that harvesting or mowing changes the scene of the lines of the field. In most of the cases, though, the plants grow in rows, producing a natural set of lines, which define the image, thus making the processing more distinct. In general, autonomous navigation systems for agricultural mobile robot consists of navigation sensors, computational methods, and navigation control strategies. Figure 1 presents the aforementioned basic elements. 

Image processing techniques have been implemented to crop row images in order to define the directrix for a vehicle. In what follows, the most recent techniques of the literature are briefly described. Table 7 summarizes the selected applied techniques and comparatively presents their reported pros and cons. The aim of this section is to suggest the deep insights and general knowledge regarding vision-based vehicle guidance systems by grouping or analyzing approaches through similar research articles of the literature. 

In [90], crop rows are identified in images using Hough transform. The platform proposed in [91] uses an E/H steering system and a feedforward-plus-PID steering controller in support of automated guidance actuation. Redundant posture sensor, including GPS, geomagnetic direction sensors (FOG), and machine vision sensing systems are used on this platform for providing automated guidance information. A robust procedure with row segmentation by K-means clustering algorithm, row detection by a moment algorithm, and guidance line selection by a cost function are proposed in [88]. Auxiliary information, such as known crop row spacing, is used for the development of the guidance directrix. In order to guide an autonomous vehicle through the field in [92] a vision-based row detection system is used based on grey-scale Hough transform on intelligently merged images. The experiment takes place in a sugar beet field in a greenhouse. A method to detect crop rows to guide agricultural robots to work in real time even under a wide range of illumination situations is described in [93]. Image pre-processing is conducted to obtain the binarization image and vertical projection method to estimate the position of the crop rows for image strips. The detection of crop rows is defined by Hough transform. The machine vision-based autonomous navigation system in [94] constitute three steps. Firstly, the camera calibration is applied to obtain the relationship between the image coordinates and the world coordinates. Then, pattern recognition and image processing are used to obtain a quasi-navigation baseline. Lastly, the real navigation line is extracted from the quasi navigation baseline via Hough transform.

A color image of an orchard is classified into orchard elements by a multilayer feedforward neural network in [95]. The extraction of the desired path is performed by Hough transform after a filtering operation. A novel vision-based technique for navigation of agricultural mobile robots in orchards is suggested in [96]. The captured color image is clustered by the mean-shift algorithm. Then, a classification technique based on graph partitioning theory classifies clustered image into defined classes including terrain, trees and sky. Hough transform is applied to extract the required features to define the desired central path for robot navigation in orchard rows. A RTK-DGPS (real time kinematic differential global positioning system)-based autonomous field navigation system including automated headland turns is presented in [97] to provide a method for crop row mapping combining machine vision, and to evaluate the benefits of a behavior-based reactive layer in a hybrid deliberate system architecture. Vision-based row guidance is presented in [98]. The camera is used to detect and identify crop plants, and then accurate and stable navigation information is extracted from the binary image, which includes the offset and heading angle of the robot relative to the guidance row from the image. The vehicle in [99] uses a combination of GPS and machine vision to navigate kiwifruit orchards, maneuver around obstacles such as posts, and recognize braces. In [84], a machine vision sensor is proposed to guide a vehicle in a row of trees. The basic concept of applying machine vision for navigation is for measuring the relative position of the vehicle concerning a landmark and use that to estimate the vehicle’s heading. The goal of [84] is to develop an algorithm to autonomously guide a small robotic ground vehicle platform along an orchard row, following the path of the row using an upward looking camera combined with a controller based on feature recognition from the contrast between the tree canopies and the sky. The method presented in [100] is based on a particle filter (PF) using a novel measurement model, where a model image is constructed from the particle and compared directly with the measurement image after elementary processing, such as down-sampling, excessive-green filtering and thresholding. Machine vision and laser radar (ladar) are individually used for guidance in [101]. A rotary encoder is used to provide feedback on the steering angle. A PID controller is developed to minimize the path error. The vehicle’s guidance accuracy is tested in flexible test paths constructed of common hay bales. A stereo image processing algorithm is developed in [102] which detects and tracks ground features captured in two consecutive field images, acquired using a vehicle-mounted stereo camera. These ground features are used as reference points to calculate the lateral offset. A precise measurement of the vehicle’s lateral offset can help achieve navigation accuracy. A variable field-of-view machine vision method is developed in [103] allowing an agricultural robot to navigate between rows in cornfields. Guidance lines are detected using an image processing algorithm, employing morphological features in a far, near and lateral field of view, and the robot was guided along these lines using fuzzy logic control. In the method presented in [104] natural frames are analyzed in RGB vector space to research the feasibility of curve path detection of unstructured roads. Perceptual color clustering and morphological image processing have been used as pre-processing to obtain the segmented path. The pixels of the segmented image are analyzed from right to left, line-by-line to find the middle points of the path. In order to obtain the optimal navigation path, the least-squares curve fitting method is implemented, and finally, a new method of obtaining the navigation parameters is introduced. In [105], the HSΙ (hue, saturation, intensity) color model is used to process images, and a threshold algorithm based on the H component is used to produce grayscale images. According to the characteristics of the crop rows in the image, a method of crop line identification based on linear scanning is proposed. Fuzzy control is used to control the agricultural implement. 

Although there have been research developments in machine vision guiding systems during the last years, shortcomings such as low robustness of versatility and dependability of technologies are obstructing the improvements required for the commercialization of guidance systems. It should be noted that GPS and machine vision technologies need to be combined or at least one of them needs to be fused with another technology (e.g., laser radar) as to develop efficient agricultural vehicle guidance systems in the future. Moreover, autonomous navigation in an agricultural environment is demanding due to the inherent uncertainty of the environment. The robot needs to navigate in an unstructured, dynamically changing environment with many sources of noise (e.g., rough terrain or varying shapes, sizes and color of the plants or inconsistency in the environment structure). This problem is enhanced by adding the hardware related noise like imprecise sensor measurements, wheel slippage, controller and actuator noise. Developing a navigation system to handle all the aforementioned uncertainties and their variations is not simple but rather challenging.

## 7. Vision-Based AgroBots

Agricultural practices have introduced a new problem, namely the need to implement guidance technology, e.g., a fertilizing machine or a row weeder along a crop row, with extreme accuracy. Typically, this was performed manually by a person having a direct view of the rows. This is getting impossible with the modern growing concept, which is demanding in terms of money and time. The narrow row spacing compared to the wide implementations proposed for agricultural practices, would require very careful handling and quick reactions by the human operator. Moreover, industrialized application in vast fields would be overwhelming for a human operator. Automatically guided agricultural vehicles will not fatigue, and can reduce an operator’s work intensity, resulting in enhanced efficiency and increased operation safety. For this reason, the need for developing systems for automatic guidance with respect to crop rows has become imperative. Agricultural robots, namely AgroBots, were introduced to apply precision techniques for agricultural operations such as harvesting, weeding, spraying, transportation, etc. Such techniques are also involved in the application of chemicals that are placed in such a way as to have an optimal effect with the minimum quantity or placement of fertilizer close to the crop so as to minimize the supply of nutrients to the weeds. The latter also comprises the recent but increasing concern of farmers about the environmental impacts of agriculture, not only regarding harsh chemicals, but also wasting water. The main goal of AgroBots is to sense targets online and to work on a plant scale with extreme accuracy. AgroBots can save labor costs, prevent workers from performing risky operations, and provide the farmer with up-to-date and precise information to assist management decisions. Thus, developing AgroBots is one of the challenges that agriculture is facing in industrialized countries, especially those derived from labor shortage and ever-increasing production costs.

Even though robots are widely used for automation in industry, it is rather uncommon to meet robots in agriculture. The industrial environment is, comparatively to agriculture fields, clean, dry, predictable and well-lit while fields provide unpredictable conditions in terms of light, weather and terrain. Moreover, industrial automation involves uniform, specific components robust for robotic manipulation, while agricultural automation deals with crops which vary in many ways; color, size shape, position, are frequently covered by foliage and are sensitive to handle. AgroBots have been introduced in structured environments (i.e., simulated plantation in laboratories), as well as different indoor (i.e., greenhouses) and outdoor agricultural environments. However, only a few vision-guided systems have been successfully developed and tested in real field trials. Table 8 summarizes some of the reported autonomous mobile ArgoBots from the literature. 

The study on ArgoBots is mainly divided in two categories. The first category is based on tractors and other agricultural machinery to develop intelligent systems with navigation functions as described in the previous section. The other category is to independently develop intelligent mobile platforms for agriculture, as described in the current section. However, the latter intelligent platforms are used to solve specific problems on automation of agricultural production, and they are limited to specific functions. In other words, they refer to specific application environments, thus, it is not easy to expand and improve. Moreover, the commercialization of AgroBots is scarce due to their limited seasonal usage, which indirectly increases the cost of agricultural production. The reliability of a machine to fulfill precise farming work instead of human is another drawback that keeps AgroBots off the market. The intelligence level of AgroBots needs to be improved further so as gain the farmer’s confidence. Though high intelligence is required for an AgroBot to achieve higher production, it is very important to be: (1) simple in its structure, (2) affordable, in terms of what it offers, (3) easy to manipulate, and (4) adaptive to the needs of the agricultural production. A simple structure refers also to a small size. A robot of a smaller size is more easily transferable and possibly less expensive. It should be investigated if crop production may be performed quicker, better, and cheaper with a swarm of small robots rather than a few large ones. Moreover, one of the advantages of smaller robots is that they may become acceptable and popular to the non-agricultural community, for domestic personal use rather than mass usage.

Table 8 reports in chronological order some of the autonomous mobile ArgoBots from the literature. The Agrobot presented in [90] uses threshold plant blobs as input features and Hough transform to find the vehicle offset and heading angle with respect to the plant rows. Small errors in offset and angle are reported. The effect of vision errors can be reduced when the data are fused with odometry in the vehicle controller. The angular accuracy could also be improved by adjusting the optical configuration to give more perspective. The vehicle of [106] is affected by the distribution of the fruits in the tree. Moreover, it is reported for the robot a mean picking time of fruit greater than that of a human picker. Modifications have been suggested in order to improve its performance, including new algorithms for real-time fruit tracking, evaluation of the fruit’s 3D position, and mechanical modifications of the structure so the arms are able to reach the fruit quickly and simultaneously. In [107], the proposed vision system appears to be very robust. The robot can navigate with acceptable behavior. Reported disadvantages are the oscillations when the vehicle comes out of the turns. Future work should include a model predictive control navigation technique in order to diminish the oscillations and more exhaustive tests on the developed system for different speed values and more difficult paths. The robot implemented in [108] demonstrates good ability to target objects with subcentimetre accuracy. However, under realistic outdoor conditions, the accuracy is reduced. Unpredictable conditions such as weed, varying soil and wind effects are considered as potential problems. Future work suggests shielding in order to minimize the potential wind effects. The agricultural robot of [99] has four picking arms, each of which is programmed to pick one fruit per second. The system is characterized by adequate vision, navigation, and the delicacy of fruit picking and handling. The system developed in [98] exhibits good performance. Preliminary experimental results on the algorithms of row identification and row guidance are effective according to the parameters measured and analyzed such as the heading angle and the offset for row guidance and the difference between the motion trajectory of the robot and the expected trajectory. Compared with the indoor experiment, the in-field experiment reveals lower performance. The main reasons for this include some factors such as a rough field, man-made measurement error, wheel sideslip due to soft and moist soil, etc. Additionally, it should be noted that the plants in the field were selected artificially with a similar size and height and planted along rows. The Agrobot of [97] can be guided along a defined path with centimeter precision. The architecture of the robot makes it easily expandable by adding new behaviors with small changes. In order to be able to navigate precisely under bumpier conditions, roll compensation is required. The vehicle of [109] is reported to generate navigation path reliably. However, the implemented algorithms are suitable for fields where the ground contains fewer weeds. and the main areas of trees are more visible. 

## 8. Discussion

In this paper, recent research efforts in machine vision for agriculture applications were reviewed. Machine vision can support agricultural activities by providing accurate and efficient solutions that have been traditionally performed manually. Manual methods tend to be labor intensive and error prone. In particular, this paper addresses three main issues concerning the machine vision systems in agricultural task of crop farming, namely (1) plant/fruit detection (segmentation), (2) harvesting, and (3) health monitoring. For each of these tasks, challenges, research gaps and potential future directions are being discussed in the rest of the section.

In order for a machine vision approach to perform an agricultural activity like fruit grading first image segmentation should be performed in order to separate the target (i.e., fruit) from the background. Therefore, plant and fruit detection are very important and critical tasks. Illumination variations, fruits overlapping, hidden fruit in foliage and branches, and similar color variations between the target and its background are some of the main factors that affect the segmentation process. The appropriate handling of these factors constitutes a great challenge towards developing more robust and accurate machine vision based agrobots. Color-based plant and fruit detection approaches are described in [15,16,19,40,41]. However, color approaches can be affected by the lighting variations. Changing lighting conditions are addressed by the methods presented in [22,35] which deploy histogram selection with mean grey value and CLAHE [27], respectively, for enhancing the robustness against various lighting conditions. Texture, color and size features are used in [21] for the detection of grains and classification of their species, while [36] uses color and shape features for segmenting cucumber leaves. The latter work [36] addressed the problem of overlapping by deploying edge detection with Sobel operators and C-V model. Lastly, the problem of similar colors between the target and background is addressed in [16], which uses super-pixels in order to generate a saliency map and Gaussian curve fitting for segmenting green apples. Regarding the used machine learning algorithms, it is worth noting that the SLIC clustering method is deployed in [17,22,29], neural networks are also applied in several studies [15,20,21,35], while [17] uses SVM and PCA for reducing the features dimensions. 

Harvesting is a main agricultural activity and refers to the collection of ripe fruit or crops. Machine vision has been deployed for supporting harvest by providing solutions on automated fruit grading, ripeness detection, fruit counting and yield prediction. Fruit grading refers to the sorting of fruits based on inherent properties like size and shape. Thus, machine vision approaches for fruit grading deploy besides color, geometrical and shape features [38,43]. Ripeness detection is mostly performed based on color [41,43]. For fruit counting, color [45,47] and morphological features [37] are deployed. Overlapping of fruit and occlusion are addressed in [45], which use circular fitting with Hough transform. The variation in lighting conditions are addressed in [37], which use an adaptive global threshold and morphological cues. As far as the use of machine learning algorithms is concerned, neural networks (convolutional and back propagation) are also deployed [46,48].

Machine vision is also used for protecting the health of the plant and detecting deficiencies and diseases. Weed detection is a main agricultural activity that is performed in order to protect the plant’s growth. Weeds share the same resources with the plant (like moisture) and thus can affect plant health. The main challenges in this task are the handling of the illumination variation and the overlapping leaves. A color-based weed detection approach in carrot fields is presented in [49]. In the case of weed detection in crop fields where the color difference is not very high, a texture-based approach can be deployed [50] to tackle the overlapping cases. Research conducted in [52] revealed that the proposed weed detection approach based on SURF features and Gaussian mixture model outperforms a geometry-based approach based on random forests [55] and a FCN, which took into account vegetation indices as features. A region-based weed detection approach is suggested in [57]. However, it cannot accurately classify the pixels of regions that contain both value crop and weed pixels. For those cases, a pixel-based classifier is proposed. Regarding the used machine learning algorithms, neural networks [49,50,54,56] and SVM [57] are commonly applied. Insects can cause damage to plants and fruits. For better protecting the plant health, vision-based approaches for insect detection have been proposed. Clustering based on color features is deployed in [61] for *L. botrana* recognition in grapevines, while SVM is deployed in approaches presented in [62,63], which consider SIFT, color, and morphological features, respectively. Disease and deficiency detection refers to the automatic detection of parts of the plant that are not healthy. Challenges related to this task include the handling of illumination variations and of the color variance of the diseased part [3]. Automatic identification of nutritional deficiencies in coffee leaves are addressed in [64]. Naïve Bayes and BSM descriptor are reported outperforming k-NN and Neural Network classifiers. However, the reported F-measure is not high (64.9%). A bagged tree ensemble classifier based on color features is proposed in [65] for detecting diseases in citrus plants. The experimental results show that the proposed classifier outperformed k-NN, SVM and boosted tree classifiers. A method for detecting lesions on wheat leaves based on the C-V model is presented in [68], while PCA and K-means clustering are applied in [69]. Experimental results reveal that this approach outperforms Otsu segmentation. Illumination variation is addressed in [72], which proposed a new color feature vector, which is invariant to illumination. The experimental results showed that the proposed approach outperforms K-means clustering and Otsu segmentation on images captured under real field conditions. Finally, SVM classifiers are deployed in research efforts [66,67,71,75,76,78].

## 9. Conclusions

Machine vision is integrated in agricultural settings for a number of different tasks. The appropriate selection of a system is an additional guarantee for the accomplishment of most agricultural operations with precision and relative speed. In this regard, this work has addressed the following main topics: (1) plant and fruit detection approaches, (2) harvesting support approaches including fruit grading, ripeness detection, fruit counting and yield prediction, (3) plant and fruit health protection, and disease detection approaches including weed, insect detection, disease and deficiency detection, (4) camera types used for machine vision in agriculture, (5) vision-based vehicle guidance systems (navigation) for agricultural applications, and (6) vision-based autonomous mobile agricultural robots. 

The design of adequate software is a key factor for the effective functioning of mobile robots. The presentation of recent algorithms for basic agricultural tasks that took place in the previous sections is in-line with this key factor; presenting the recent trends on machine vision algorithms for basic agricultural tasks in order to be evaluated, selected and integrated to a mobile robot. Algorithms need to be suitable for the automation of agricultural operations oriented for a semi-structured environment, such as an agricultural environment. That is, some structures within the fields are known, e.g., the columns in a grapevine or the exact position of the trees in an olive grove, so deterministic tasks related to them can be optimized. However, a field is more of a non-stable environment. Reactive tasks that take place in the fields need to be executed in real-time and have to consider dynamic local conditions, impossible to be known a priori (weed, foliage, bad weather condition, lighting etc.). On top of that, researchers need to develop appropriate robot architectures that could coordinate reactive behaviors, like stopping on emergencies or reacting to specific inputs in a decision-making way, thus leading to autonomous intelligent robotic behaviors in the field. 

The aim of this work is to serve as a guide for the researcher and practitioner alike in the agricultural field by providing the latest advancements in machine vision applications for agriculture with a focus on crop farming. To this end, the current review does not only focus on a specific agricultural challenge (e.g., disease detection) or fruit type or plant (e.g., citrus) but rather outlines research conducted in different agricultural activities of crop farming, also covering more technical subjects, such as vision-based vehicle guidance systems and autonomous mobile agricultural robots.

## Figures and Tables

**Table 1:** Plant/fruit detection approaches.

Ref.	Fruit/Plant Type	Task	Feature Type	Method	Performance Indices
[16]	Green apples	Detection of green apples in natural scenes	Color (V-component of the YUV color space)	Saliency map and Gaussian curve fitting	Accuracy = 91.84%
[17]	Apples	Apple flower detection	Color and spatial proximity	SLIC + CNN SVM + PCA	AUC-PR and F-measure 93.40% on Apple A dataset F-measure 80.20% on Apple B F-measure 82.20% on Apple C F-measure 79.90% on Peach
[15]	Grapes	Grape identification	Color (HSV and L* a* b* color spaces)	ANN+GA	Accuracy = 99.40%
[19]	Mango	Mango leaf image segmentation	Color (HSV and YCbCr color spaces)	Otsu	Precision = 99.50% Recall = 97.10% F-measure = 98.30%
[20]	Plants of 6 different types e.g., Potato, Mallow.	Segmentation of different plants in different growth stages, different conditions of the day and one controlled state and different imaging situations	Color (Five features among 126 extracting features of five colour spaces RGB, CMY, HSI, HSV, YIQ and YCbCr were selected using a hybrid ANN)	ANN-HS	Accuracy = 99.69%
[21]	Grains	Classification of wheat grains to their species	Size, color and texture	Otsu + MLP	Accuracy = 99.92%
[22]	Flower and seedpods of soybean plants	Flower and seedpods detection in a crowd of soybean plants	Color (Hue of HSV color space) for flower detection. Haar-like features for seedpods detection	SLIC + CNN Viola-Jones detector + CLAHE + CNN	Precision = 90.00% Recall = 82.60% F-measure = 84.00%
[29]	Rice	Image segmentation for rice panicles	Color (L, a, b values of the CIELAB color space)	SLIC + CNN + ERS	F-measure = 76.73%
[35]	Cotton	Cotton leaves segmentation	Color (three anti-light color components were selected by histogram statistical with mean gray value)	PCNN + Immune algorithm	Accuracy = 93.50%
[36]	Overlapping leaves (tested on cucumber leaves)	Image segmentation of overlapping leaves	Color and Shape	Sobel + C-V model	Accuracy = 95.72%

**Table 2:** Fruit grading and ripeness detection approaches.

Ref.	Fruit Type	Task	Feature Type	Method	Performance Indices
[38]	Mango	Grading based on size (classifying mangos into one of three mass grades, e.g., large, medium, and small)	Color, Geometrical and Shape	Region based global thresholding color binarization, combined with median filter and morphological analysis	Accuracy = 97.00%
[39]	Mango	Size estimation	Histogram of Oriented Gradients (HOG), Color (CIE L*a*b* color space)	Cascade classifier, Otsu thresholding & ellipse fitting method	Precision 100% on fruit detection. R2 = 0.96 and RMSE = 4.9 mm for fruit length estimation and R2 = 0.95 and RMSE = 4.3 mm for fruit width estimation
[40]	Olive	Estimation of the maximum/minimum (polar/equatorial) diameter length and mass of olive fruits	HSV color space (the value and saturation channels) & morphological features	Mathematical morphology & automated thresholding based on statistical bimodal analysis	Relative mean errors below 2.50% for all tested cases
[41]	Tomato	Ripeness detection	Color (HSI color model)	BPNN	Accuracy = 99.31%
[43]	Citrus	Ripeness detection	Color & shape	Circular Hough Transform + CHOI’s Circle Estimation (‘CHOICE’) + AlexNet	Recall = 91.60%, 96.00% and 90.60 for, NIR and depth images respectively

**Table 3:** Fruit counting and yield prediction approaches.

Ref.	Fruit/Plant Type	Task	Feature Type	Method	Performance Indices
[49]	Marigold flowers	Detection and counting	Color (HSV color space)	HSV color transform and circular Hough transform (CHT)	Average error of 5%
[37]	Tomato flowers	Detection and counting	Color (HSV) and morphological (size & location)	Adaptive global threshold, segmentation over the HSV color space, and morphological cues.	Precision = 80.00% Recall = 80.00%
[46]	Apples and oranges	Fruit detection and counting	Pixel based	CNN and linear regression	Mean error (in the form of ratio of total fruit counted and standard deviation of the errors) of 13.8 on the oranges, and 10.5 on the apples.
[48]	Apples	Early yield prediction	Colour (RGB & HIS) and Tree canopy features	Otsu + BPNN	R^2^ 0.82 & 0.80
[47]	Citrus	Detection and counting	Color (HSV)	Histogram thresholding + Watershed segmentation	Mean of the absolute error 5.76%

**Table 4:** Weed detection approaches.

Ref.	Fruit/Plant Type	Task	Feature Type	Method	Performance Indices
[61]	Weed in cauliflower plants fields	Weed detection	Color (HSV color space) and morphological	Morphological Image Analysis	Recall = 98.91% Precision = 99.04%
[50]	Weed in carrots fields	Weed detection	Color (HSV color space)	CNN	Precision = 99.10% F-measure = 99.60% Accuracy = 99.30%
[53]	Broad-leaved dock weed	Weed detection	Speed-Up Robust Feature (SURF) [54],	SURF + GMM	Accuracy = 89.09% and false-positive rate 4.38%
[57]	Weed in crop fields	Weed detection	Color (RGB) and plant features (vegetation indices)	Deep encoder-decoder CNN	Results in 3 datasets: Precision 98.16% & Recall 93.35%, Precision 67.91% & Recall 63.33%, Precision 87.87 & Recall 64.66%
[51]	Weed in sugarbeet fields	Weed detection	Wavelet texture features	PCA+ANN	Accuracy = 96.00%
[55]	Weed in sugarbeet fields	Weed detection	Spatial information	CNN + encoder-decoder structure	Results in 2 datasets: Precision 97.90% & Recall 87.80%, Precision 72.70% & Recall 95.30%
[58]	Weed in sugarbeet fields	Weed detection	Attribute morphology based	Selection of regions based on max-tree hierarchy [59] and classification with SVM	F-measure = 85.00% (onions) F-measure = 76.00% (sugarbeet)

**Table 5:** Insect detection approaches.

Ref.	Fruit/Plant Type	Task	Feature Type	Method	Performance Indices
[62]	Grapes	L. botrana recognition	Color (gray scale values and gradient)	Clustering	Specificity = 95.10%
[63]	Grapes	Grapevine bug detection	SIFT	SVM	Precision = 86.00%
[64]	Strawberry plants	Pest detection in strawberry plants	Color (HSI color space) & morphological (ratio of major diameter to minor diameter in region)	SVM	MSE = 0.471

**Table 6:** Disease and deficiencies detection approaches.

Ref.	Fruit/Plant Type	Task	Feature Type	Method	Performance Indices
[65]	Coffee leaves	Identification of nutritional deficiencies of Boron (B), Calcium (Ca), Iron (Fe) and Potassium (K)	Color, shape & texture	Otsu + BSM + GLCM	F-measure = 64.90%
[68]	Plants	Plant disease detection	SIFT+ statistical features	SVM	Accuracy = 78.70%
[70]	Wheat	Detecting lesions on wheat leaves	Color	(C–V) + PCA + K-means	Accuracy = 84.17%
[71]	Soybean leaves	Image retrieving diseased leaves of soybean	HSV Color + SIFT + LGGP	Calculation of distance of the feature vectors to find the one with the smallest distance.	Average retrieval efficiency of 80.00% (for top 5 retrieval) and 72.00% (for top 10 retrieval)
[72]	Potato leaves	Disease detection	Color, texture and statistical	Hard thresholding (L*, a* and b* channels) + SVM	Accuracy = 93.70%
[72]	Greenhouse vegetable	Detection of foliar disease spots	CCF + ExR	Interactive region growing method based on the CCF map	Precision = 97.29%
[66]	Citrus plants	Disease detection	Color (RGB, HSV color histogram) and texture (LBP)	Delta E (DE) + Bagged tree ensemble classifier	Accuracy = 99.90%
[75]	Citrus leaves	Disease detection and classification	GLCM	K-means + SVM	Accuracy = 90.00%
[76]	Citrus leaves	Disease detection and classification	Color, texture, and geometric	PCA + Multi-Class SVM.	Accuracy = 97.00%, 89.00%, 90.40% on three datasets
[77]	Cucumber leaves	Disease detection and classification	Shape and color	K-means + SR	Accuracy = 85.70%
[78]	Cucumber and apple leaves	Disease detection and classification	PHOG [80]	K-means + Context-Aware SVM	Accuracy = 85.64% (apples) Accuracy = 87.55% (cucumber)
[67]	Apple leaves	Disease identification	Color, texture and shape features	SVM	Accuracy = 94.22%

**Table 7:** Machine vision-based techniques for automated navigation of agricultural vehicles.

Ref.	Reported Advantages	Reported Disadvantages	Main Technique
[90]	Integrates information over a number of crop rows, making the technique tolerant to missing plants and weeds.	Confined to situations where plants are established in rows.	Hough transform
[91]	Points in various directrix classes were determined by unsupervised classification. The sensor fusion-based navigation could provide satisfactory agricultural vehicle guidance even while losing individual navigation signals such as image of the path, or the GPS signal for short periods of time.	When the steering input frequency was higher than 4 Hz, the steering system could not respond to the steering command.	A heuristic method and Hough transform
[88]	Good accuracy from two test data sets: a set of soybean images and a set of corn images. The procedure is considered acceptable for real-time vision guidance applications in terms of its accuracy.	K-means algorithm in the procedure limits the processing speed, which is acceptable for real-time applications if the controller output rate is faster and is independent of the image update rate. The performance of the image processing procedure degrades significantly under adverse environmental conditions such as weeds. The fusion of vision sensor with other navigation sensors, such as GPS, is needed in order to provide a more robust guidance directrix.	K-means clustering algorithm, a moment algorithm, and a cost function
[92]	A considerable improvement of the speed of image processing. The algorithm is able to find the row at various growth stages.	Inaccuracies exist because of a limited number and size of crop plants, overexposure of the camera, and the presence of green algae due to the use of a greenhouse. Inaccuracies accounted for by footprints indicate that linear structures in the soil surface might create problems. High image acquisition speed, results to images overlapped each other for a large part, thus, total amount of images is too large.	Grey-scale Hough transform
[93]	In the pre-processing the binarization image was divided into several row segments, which created less data points while still reserved information of crop rows. Less complex data facilitated Hough transform to meet the real-time requirements.	Rows and inter-row spaces could not be segmented clearly. Within the growth of wheat, the rows became overlapped. Narrow inter-row spaces of the field made it difficult to discriminate all of the rows in view. A great number of weeds between the crop rows disturbed the row detection algorithm	Vertical projection method and Hough transform
[94]	The system is not restricted to a specific crop and has been tested on several green crops. After examination in a laboratory and in-field, results show that the method can attain navigation parameters in real time and guide the mobile robot effectively.	Camera calibration has good results if the robot moves in a smooth field. When the field is uneven, the calibration result is unstabilized. Rows and inter-row spaces must be segmented clearly so as the quasi navigation baseline to be detected easily. If the field has narrow inter-row spaces, rows became overlapped, which makes them difficult to separate from background.	Grey-level transformation, Otsu binarization, and Hough transform
[95]	Achieves good classification accuracy. The desired path is properly calculated in different lighting conditions.	Classification based on NN requires training, validation and testing of a number of samples, which is time-consuming. NN classification is a supervised technique.	A multilayer feedforward neural network and Hough transform
[96]	The proposed technique is unsupervised. It can be used as a complementary system to the other navigational systems such as LiDAR to provide a robust and reliable navigation system for mobile robots in orchards.	For not well-structured orchards, a filtering process is required to extract the path. Not tested in different lighting conditions.	Mean-shift algorithm, graph partitioning theory, and Hough transform
[97]	The method limits required a priori information regarding the field. Paths are generated in real-time. The architecture of the software is transparent, and the software is easily extendible.	Very slow. Further improvements in the accuracy of path following of a straight path are not to be expected due to technical reasons.	Grey-scale Hough transform
[98]	A local small window of interest is determined as the ladder structure to improve the real-time detection algorithm, and to minimize the effect of the useless information and noises on detection and identification.	Error of the camera position would result in errors of the provided data. Therefore, the camera requires efficient calibration. The plants in the field were selected artificially with the similar size and height and planted in a perfect row. Easily affected in real field conditions, e.g., uneven field, man-made measure error, wheels sideslip due to soft/moist soil etc.	Edge detection, image binarization, and least square methods
[99]	The robot has demonstrated capability to use artificial vision for navigation without artificial visual markers. The vision software has enough intelligence to perceive obstacles.	When the vehicle is under the canopy, it relies on the cameras to find the way, but when it is not under the kiwifruit canopy the system relies on GPS and a compass to navigate. Because the camera lenses are short, provision must be made to handle fisheye in the stereopsis.	Hough transform
[84]	Inconsistent lighting, shadows, and color similarities in features are eliminated by using a sky-based approach where the image was reduced to canopy plus sky, thus simplifying the segmentation process. This produces a more sensitive control system. The cropped image contains less data needing to be processed, resulting in faster processing time and more rapid response of the ground vehicle platform.	There are large deviations from the center of the row when there are sections with a break in the canopy due to either a missing tree or a tree with limited leaf growth. The proposed approach is effective only when the trees have fully developed canopies. Inadequate for canopies year-round such as citrus or fruit trees that lose their leaves in the winter. It only tackles the straight-line motion down the row but not the end of the row conditions.	Thresholding approach, filtering, and determination of centroid of the path
[100]	The proposed model does not extract features from the image and thus does not suffer from errors related to feature extraction process. Efficient for different row patterns, varying plant sizes and diverse lighting conditions. The robot navigates through the corridor without touching the plant stems, detects the end of the rows and traverses the headland	Lack of robustness to uncertainties of the environment. The estimation of the state vector is less accurate during headland compared to row following. When the robot is in the headland only a small part of the rows is visible making the estimation of the orientation of the robot heading less precise. Incorrect estimates of row width and row distance result to inaccuracies.	Particle filter algorithm
[101]	Alternative navigation method where the tree canopy frequently blocks the satellite signals to the GPS receiver. The performance is better at lower speeds of the vehicle.	No obstacle detection capability. Low performance at high speed of the vehicle.	Segmentation algorithm
[102]	Successfully tested on both straight and curved paths, navigation is based only on stereoscopic vision.	Reduced accuracy of lateral offset estimation when the vehicle navigates on curved paths. The algorithm is developed based on the assumption of no camera rotations while travelling in the field. Not implemented in real-time.	Stereo image processing algorithm
[103]	Acceptable accuracy and stability, tested without damaging the crop.	Uneven cornfield caused by residual roots and hard soil clods results in a poorer performance.	Segmentation and Max–Min fuzzy inference algorithm
[104]	High speed and remarkable precision. The navigation parameters in the algorithm can control the robot movement well. The algorithm displays better overall robustness and global optimization for detecting the navigation path compared to Hough transform, especially in curved paths.	When using a straight line to detect navigation directrix, the algorithm does not perform well.	Image segmentation, and least-squares curve fitting method
[105]	Resolves the problem of illumination interference for image segmentation, adapts to changes in natural light and has good dynamic performance at all speeds.	No obstacle avoidance	Threshold algorithm, linear scanning, and a fuzzy controller

**Table 8:** Vision-based autonomous mobile AgroBots.

Ref.	Description	Application (Year)	Photo
[90]	Visual sensing for an autonomous crop protection robot. Image analysis to derive guidance information and to differentiate between plants, weed and soil.	Tested in cauliflowers, sugar beet and widely spaced double rows of wheat (1996)	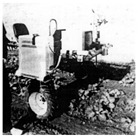 Image from [90]
[106]	A commercial agricultural manipulation for fruit picking and handling without human intervention. Machine vision takes place with segmentation of a frame during the picking phase.	Semi-autonomous orange picking robot (2005)	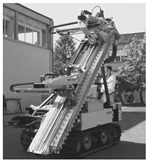 Image from [106]
[107]	Use of a vision system that retrieves a map of the plantation rows, within which the robot must navigate. After obtaining the map of the rows, the robot traces the route to follow (the center line between the rows) and follows it using a navigation system	Tested in a simulated plantation and in a real one at the “Field robot Event 2006” competition (2006)	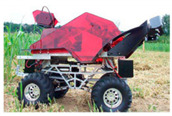 Image from [107]
[108]	Precise application of herbicides in a seed line. A machine vision system recognizes objects to be sprayed and a micro-dosing system targets very small doses of liquid at the detected objects, while the autonomous vehicle takes care of the navigation.	Experiments carried out under controlled indoor conditions (2007)	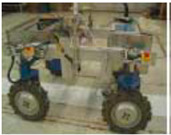 Image from [108]
[99]	The vision system identifies fruit hanging from the canopy, discriminating size and defects. Fruits are placed optimally in a bin and when it is full, the vehicle puts the bin down, takes an empty bin, picks it up and resumes from its last position	Autonomous kiwifruit-picking robot (2009)	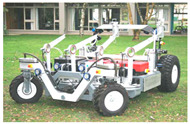 Image from [99]
[98]	The offset and heading angle of the robot platform are detected in real time to guide the platform on the basis of recognition of a crop row using machine vision	Robot platform that navigates independently. Tested in a small vegetable field (2010)	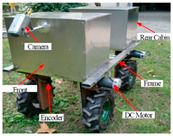 Image from [98]
[97]	Development of an RTK-DGPS-based autonomous field navigation system including automated headland turns and of a method for crop row mapping combining machine vision and RTK-DGPS.	Autonomous weed control in a sugar beet field (2011)	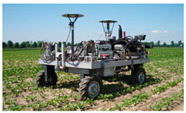 Image from [97]
[109]	An algorithm of generating navigation paths for harvesting robots based on machine vision and GPS navigation.	Tested in an orchard for apple picking (2011)	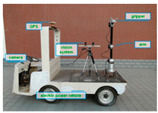 Image from [109]
